# The correlation between serum albumin and diabetic retinopathy among people with type 2 diabetes mellitus: NHANES 2011–2020

**DOI:** 10.1371/journal.pone.0270019

**Published:** 2022-06-16

**Authors:** Gao-Xiang Wang, Ze-Bin Fang, Jun-Tong Li, Bao-Li Huang, De-Liang Liu, Shu-Fang Chu, Hui-Lin Li

**Affiliations:** 1 Shenzhen Traditional Chinese Medicine Hospital Affiliated to Nanjing University of Chinese Medicine, Shenzhen, Guangdong, China; 2 Department of Endocrinology, Shenzhen Traditional Chinese Medicine Hospital, Shenzhen, Guangdong, China; 3 The Fourth Clinical Medical College of Guangzhou University of Chinese Medicine, Shenzhen, Guangdong, China; GSVM Medical College, INDIA

## Abstract

**Objectives:**

The objective of this research aimed to investigate the correlation involving serum albumin with diabetic retinopathy (DR) in patients with type 2 diabetes mellitus (T2DM).

**Methods:**

From 2011 to 2020, the National Health and Nutrition Examination Survey (NHANES) surveyed 45462 participants. We used the relevant data to conduct descriptive statistics, linear regression, and Logistic regression analysis.

**Results:**

After adjusting for age, sex, and race, as well as all other variables, serum albumin was significantly negatively related to DR (P<0.001). Furthermore, after controlling for confounding factors, the third quartile (Q3) and the fourth quartile (Q4) had quite a negative significant relationship with the incidence of DR (P<0.01). The second quartile had a significant positive correlation with DR, whereas the observed negative correlations were not statistically meaningful (P>0.05).

**Conclusion:**

Albumin levels in the serum have a quantitatively significant negative correlation with DR. Serum albumin levels in the blood can be used as a reference point for protracted follow-up of people with T2DM.

## 1. Introduction

As per the International Diabetes Federation (IDF), 454 million people worldwide will be living with diabetes mellitus (DM) in 2030, approximately 700 million in 2045 [1]. The prevalence of diabetic retinopathy (DR) individuals internationally is expected to hit 160.5 million by 2045, according to the latest analysis [2]. DR is the fifth most common leading cause of blindness in individuals over 50 on a worldwide scale [3]. Previously, hypertension, higher HbA1c levels, and duration of diabetes, among other chronic diseases, were implicated in the pathogenesis of DR [4].

DR is associated with chronic and low–grade inflammation, oxidative stress, and microvascular changes in neurodegeneration [5]. The neutrophil extracellular trap (NET) is a unique form of cell death. Previous studies have reported that neutrophils can contribute to DR by mediating central venous thrombosis through NET–dependent mechanisms [6–8]. Recently, a study revealed that serum albumin, a fundamental serum constituent, may help curb NET release by inhibiting the formation of IFN-β and mitochondrial ROS specifically [9]. Although numerous investigations have demonstrated that hypoalbuminemia is connected with inflammation, the correlation between serum albumin and diabetic retinopathy among people with type 2 diabetes mellitus (T2DM) is still unknow [10, 11]. Throughout this investigation, we shall investigate the correlation involving serum albumin and DR in patients with T2DM.

## 2. Methods

### 2.1 Population research

Data from the 2011–2020 National Health and Nutrition Examination Survey served as the basis for our cross-sectional study (NHANES). The poll is aimed at the general public in the United States. All participants were subjected to a battery of examinations, including height, weight, waist circumference, laboratory testing, and standardized questionnaires on education, socioeconomic status, and other factors. These data were utilized to assess the prevalence and severity of many diseases, as well as to develop public health policies and offer medical care.

### 2.2 Inclusion and exclusion criteria

**Fig 1:**
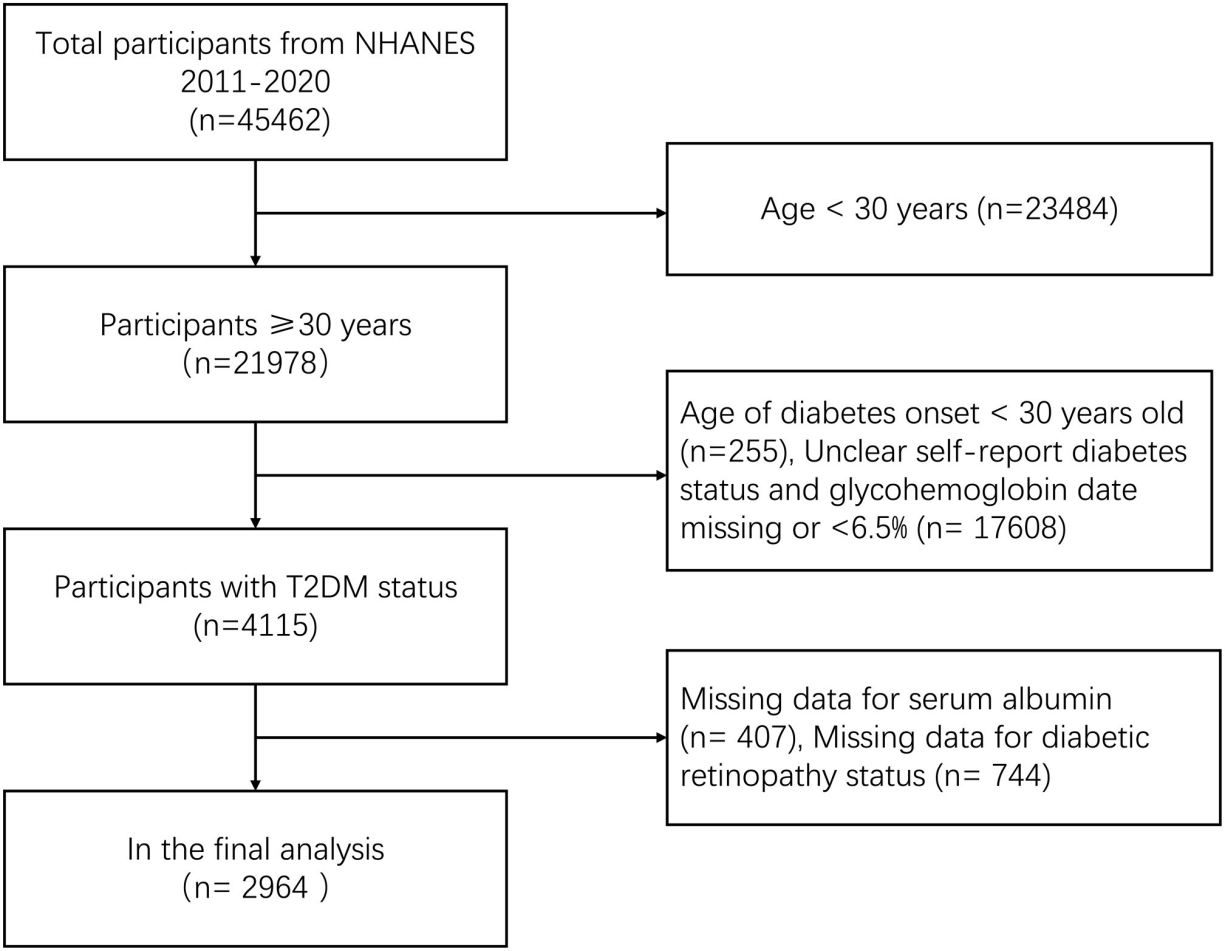
Flowchart of study target population NHANES (2011–2020).

Our study population was mainly over 30 years of age. During the study period from 2011 to 2020, we included 45,462 participants. We excluded patients under the age of 30 (n = 23,484), as well as unclear diabetes status and glycated hemoglobin data missing or < 6.5% (n = 17608). It is well known that type 1 diabetes is prevalent in people younger than 30 years old. To even further lower the probability of type 1 diabetes, people with diabetes onset before the age of 30 years (n = 255) were also removed. In addition, missing data on serum albumin (n = 407) and missing data on diabetic retinopathy (n = 744) were excluded. After screening, we included data from 2964 people in our study (Fig 1).

### 2.3 Ethics statement

A protocol for the NHANES was approved by the National Center for Health Statistics Research Ethics Review Board, and informed consent was obtained. The NHANES data is available to the public after anonymization. In this way, researchers can convert data into a study-ready format. We agree to abide by the study’s data usage guidelines to ensure that all data are used for statistical analysis and that all experiments are conducted according to applicable standards and laws.

### 2.4 DR status

The American Diabetes Association’s recommendations [12] defined T2DM as individuals informed by a practitioner that they had DM with a diagnostic age of ≥30 years or individuals with an HbA1c of ≥6.5% who did not self–report experiencing DM. Retina screening was performed on participants using professional ophthalmologists in accordance with nonmydriatic fundus photography norms.

### 2.5 Covariates

Self–reported completed a questionnaire on age, race, education level, and smoking. The doctor calculated BMI and waist circumference based on height and weight. During the study visit, information on serum albumin, total cholesterol, triglycerides, serum glucose, and glycohemoglobin was measured. Details of DR status and serum albumin. In addition, the NHANES site (http://www.cdc.gov/nchs/nhanes/) provides information on various variables gathering techniques.

### 2.6 Statistical analysis

This investigation employed the independent t-test and the chi-square test to evaluate whether there were any substantial differences between categorical and continuous variables. We utilized a multivariable logistic regression model with odds ratios (OR) and matching 95% confidence intervals (CI) to determine the correlation between different variables. Three models were constructed in conformity with the provisions of STROBE (Strengthening the Reporting of Observational Studies in Epidemiology). Model 1 was not altered in any way. Model 2 has been adjusted to account for demographic factors such as age, gender, and ethnicity. Model 3 takes into account all variables.

## 3. Results

### 3.1 Participant characterization description

Our study included 2964 individuals; 603 had diabetic retinopathy, and 2361 did not have diabetic retinopathy. Table 1 summarizes the demographic characteristics of participants with and without DR. Compared to non-DR participants, DR was significantly higher in age, blood glucose, glycosylated hemoglobin, and serum albumin, markedly lower in education, and lower in Caucasian, Black, and Mexican–Americans.

**Table 1:** Characteristics of study sample with and without diabetic retinopathy.

	Non–Diabetic Retinopathy (n = 2361)	Diabetic Retinopathy (n = 603)	P–value
Age, years	62.7874 ± 11.5870	64.3300 ± 11.0801	**0.006**
Sex, n (%)			0.375
Male	1268 (53.7061%)	336 (55.7214%)	
Female	1093 (46.2939%)	267 (44.2786%)	
Race, n (%)			**0.013**
White	782 (33.1216%)	168 (27.8607%)	
Black	631 (26.7260%)	163 (27.0315%)	
Mexican American	387 (16.3914%)	94 (15.5887%)	
Other Race	561 (23.7611%)	178 (29.5191%)	
Educational level, n (%)			**0.002**
Less than high school	696 (29.4790%)	217 (35.9867%)	
High school	542 (22.9564%)	147 (24.3781%)	
More than high school	1118 (47.3528%)	239 (39.6352%)	
Don’t Know	5 (0.2118%)	0 (0.0000%)	
Smoked at least 100 cigarettes in life, n (%)			0.581
No	1181 (50.0212%)	301 (49.9171%)	
Yes	1179 (49.9365%)	301 (49.9171%)	
Don’t Know	1 (0.0424%)	1 (0.1658%)	
BMI (kg/m2)	32.5650 ± 7.4792	32.2294 ± 7.7321	0.158
Waist circumference (cm)	110.0521 ± 15.3899	109.1813 ± 15.6134	0.111
Serum glucose (mg/dl)	150.1381 ± 70.1397	164.4909 ± 84.3491	**<0.001**
Glycohemoglobin (%)	7.3907 ± 1.7384	7.7789 ± 1.8608	**<0.001**
Triglyceride (mg/dL)	186.2664 ± 185.9530	185.0066 ± 129.6927	0.936
Total-cholesterol (mg/dL)	176.6350 ± 44.6577	174.9104 ± 47.9890	0.134
Serum Albumin (g/dL)	4.0969 ± 0.3579	4.0041 ± 0.4080	**<0.001**

Continuous variables are presented as Mean ± SD, P-value was calculated by a linear regression model. Categorical variables are presented as %, P-value was calculated by chi-square test.

### 3.2 Association between serum albumin and DR

Model 1 does not make any changes to the original design. Model 2 is one of these examples. All primary factors except age, gender, and race were considered in models 2 and 3, whereas all primary factors were taken into consideration in model 3 (Table 2). According to the results, there must have been a statistically significant negative relationship involving serum albumin and diabetic retinopathy in three categories of the regression model.

**Table 2:** Association between serum albumin (g/dl) and diabetic retinopathy status.

	Model 1, β (95% CI, P)	Model 2, β (95% CI, P)	Model 3, β (95% CI, P)
Serum Albumin	0.5166 (0.4073, 0.6552) <0.001	0.4987 (0.3912, 0.6358) <0.001	0.5253 (0.4072, 0.6777) <0.001
Quintiles of Serum Albumin			
Lowest quintile (2.0–3.8g/dL)	Reference	Reference	Reference
Q2 (3.9–4.0g/dL)	0.9689 (0.7527, 1.2472) 0.806	0.9625 (0.7458, 1.2422) 0.769	0.9991 (0.7715, 1.2938) 0.994
Q3 (4.1–4.2g/dL)	0.6614 (0.5114, 0.8553) 0.002	0.6478 (0.4994, 0.8403) 0.001	0.6703 (0.5136, 0.8748) 0.003
Q4 (4.3–5.4g/dL)	0.6057 (0.4750, 0.7722) <0.001	0.5801 (0.4519, 0.7447) <0.001	0.6164 (0.4749, 0.8001) <0.001
P for trend	**P<0.001**	**P<0.001**	**P<0.001**

Model 1: no modification variables;

Model 2: only adjusts for age, gender, and race;

Model 3: adjusts all factors

*The model is not adjusted for the stratification variable itself in the subgroup analysis.

### 3.3 Relationship between different serum albumin quartiles and DR status

The first quartile is 2–3.8 g/dl, the second quartile is 3.9–4 g/dl, the third quartile is 4.1–4.2 g/dl, and the fourth quartile digits vary from 4.3 to 5.4 g/dl, depending on the specific serum albumin levels. The negative correlation between serum albumin and diabetic retinopathy was statistically meaningful within those three models. In model 1, no characteristics were adjusted; in model 2, only age, sex, and ethnicity were modified; and then in model 3, all covariates were corrected. There was a substantial negative correlation between protein and DR in the Q3 and Q4 groups. However, no such link was observed in the Q2 group. Model 1 had a statistically significant difference between the groups, and similarly, models 2 and 3 had a statistically significant difference(Table 2).

### 3.4 Subgroup analyses

The connection between serum albumin and DR remained significant after controlling for all variables, which was confirmed in the majority of subgroup stratified analyses by age, sex, and race. In the fully adjusted model, compared with those non–DR, there were no significant associations in the 30–44 year group (OR = 1.03, 95% CI: 0.284–3.732, P = 0.965). However, in the patients’ 45–59 year group and ≥60 year group, there were significant negative associations (P <0.015, P <0.001). When the data were stratified by gender, serum albumin was found to be strongly negatively linked with DR in both males and females (P < 0.01). When stratified by race, serum albumin and DR were not significantly associated in Mexican Americans (OR = 0.539, 95% CI: 0.280–01.36, P = 0.064); significant negative associations were found in non–Hispanic whites, non–Hispanic blacks, and other races (OR = 0.542, 95% CI: 0.326–0.901, P = 0.018; OR = 0.416, 95% CI: 0.257–0.675, P < 0.001; OR = 0.591, 95% CI: 0.368–0.951, P = 0.030) (Table 3).

**Table 3:** Asociation between serum albumin(g/dl) and stratified by age, sex, and race.

	Model 1, β (95% CI, P)	Model 2, β (95% CI, P)	Model 3, β (95% CI, P)
Stratified by Age			
30–44 years	1.4795 (0.5051, 4.3333) 0.475	1.0663 (0.3410, 3.3340) 0.912	1.0295 (0.2840, 3.7315) 0.965
45–59 years	0.5494 (0.3542, 0.8523) 0.008	0.5041 (0.3208, 0.7921) 0.003	0.5516 (0.3414, 0.8913) 0.015
≥60 years	0.4701 (0.3504, 0.6306) <0.001	0.4508 (0.3344, 0.6078) <0.001	0.4673 (0.3414, 0.6395) <0.001
Stratified by Sex			
Male	0.4836 (0.3343, 0.6995) <0.001	0.5186 (0.3765, 0.7144) <0.001	0.5126 (0.3676, 0.7147) <0.001
Female	0.5034 (0.3962, 0.6398) <0.001	0.4537 (0.3122, 0.6592) <0.001	0.5211 (0.3497, 0.7767) 0.001
Stratified by Race			
Non–Hispanic White	0.5009 (0.3118, 0.8048) 0.004	0.5014 (0.3103, 0.8103) 0.005	0.5417 (0.3255, 0.9014) 0.018
Non–Hispanic Black	0.4428 (0.2804, 0.6993) <0.001	0.4272 (0.2691, 0.6781) <0.001	0.4161 (0.2566, 0.6747) <0.001
Mexican American	0.5003 (0.2738, 0.9139) 0.024	0.5141 (0.2784, 0.9493) 0.033	0.5385 (0.2798, 1.0362) 0.064
Other Race	0.5702 (0.3701, 0.8785) 0.011	0.5141 (0.2784, 0.9493) 0.033	0.5913 (0.3677, 0.9507) 0.030

Model 1: no modification variables;

Model 2: only adjusts for age, gender, and race;

Model 3: adjusts all factors

## 4. Discussion

The primary objective of this study was to evaluate whether there was a relationship between serum albumin and DR among people with type 2 diabetes. The survey participants were adults over 30 who lived in the United States and filled in key questions. In our investigation, serum albumin levels were observed to have a substantial negative connection with DR. Notably, it’s the first to demonstrate a correlation between serum albumin and DR. This significant correlation can still be observed when we adjust for age, sex, race, or all covariates. This model remains significant when stratified by age, sex, and ethnicity, except for the 30–44 and Mexican–American cohorts.

Following the publication of previous research, it was revealed that serum albumin was substantially related to diabetes, and this association remained significant even after correcting for other covariates [10]. Cross-sectional data from China showed that after controlling for other factors, multivariate logistic regression found that serum albumin was linked to DR, even when other factors were taken into account [11]. These are consistent with our findings. It’s unique to divide serum albumin levels into quartiles in the present study.

Chronic inflammation, retinal neurodegeneration, and oxidative stress may all be pathogenic variables in DR, which have long been characterized as a microvascular diseases. Many risk factors, including greater hyperglycemia, glycemic variability, duration of diabetes, hypertension, nephropathy, dyslipidemia, smoking, and BMI, can be reported for prediction [13–17]. Research has found that the early stages of diabetic retinopathy are associated with retinal vasodilation and altered blood flow velocity [5, 18]. Similarly, Sayuri Fujioka and colleagues divided the patients into two groups based on their serum Alb concentration: Group 1 had a serum Alb concentration higher than 3.8 g/dl, whereas Group 2 had a serum Alb concentration below 3.8 g/dl. Regarding the findings, Sayuri hypothesizes that the increase in blood flow velocity of the central retinal artery and central retinal vein in group 2 is due to optic disc edema caused by reduced serum albumin levels; the high blood flow velocity in group 2 is proposed to be caused by low serum albumin levels [19]. Increased oxidative stress is associated with worsening glycemic control and the advancement of DR [20]. As a metabolic outcome factor caused by sustained hyperglycemia, advanced glycation end products (AGEs) activate NF–kB by interactions with specific cellular receptors, thereby inducing cellular oxidative stress [21, 22]. In a quantitative, retrospective study, Arik Sheinenzon confirmed a negative correlation between serum albumin and CRP by linear regression analysis (r = 0.311) [23]. On the one hand, inflammation can increase the permeability of capillaries and allow serum albumin to escape. On the other hand, it can shorten albumin’s half-life and decrease its amount, resulting in hypoalbuminemia. Additionally, serum albumin factors are directly related to inflammatory reactions, quality of life, and life expectancy [24].

Although the sample presented in the study is good enough to prove the hypothesis, the current study has some limitations. Firstly, this is a cross-sectional study design. The causal relationship between serum albumin and the risk of DR could not be determined. Second, too many factors affect serum albumin levels, such as chronic liver disease, malnutrition, infections, and cancer, which may alter the experimental results. Third, our study did not stratify the risk of DR to analyze the association with serum albumin. Finally, the NHANES did not include institutionalized individuals.

## 5. Conclusions

In our research, the serum albumin–DR connection was examined. After adjusting for various potential confounders, the presence of DR was shown to be inversely linked with the level of serum albumin. When it comes to long-term monitoring of diabetes patients, the measurement of blood albumin levels can be considered as a reference. However, to better explain the mechanism of action of serum albumin and DR, further research is necessary.
